# Strengths and Challenges of Secretory Ribonucleases as AntiTumor Agents

**DOI:** 10.3390/pharmaceutics13010082

**Published:** 2021-01-09

**Authors:** Jessica Castro, Marc Ribó, Maria Vilanova, Antoni Benito

**Affiliations:** 1 Laboratori d’Enginyeria de Proteïnes, Departament de Biologia, Facultat de Ciències, Universitat de Girona, Campus de Montilivi, Carrer Maria Aurèlia Capmany, 40, 17003 Girona, Spain; jessica.castro@udg.edu (J.C.); marc.ribo@udg.edu (M.R.); 2 Institut d’Investigació Biomèdica de Girona Josep Trueta, (IdIBGi), Hospital de Santa Caterina, Carrer del Dr. Castany, s/n, 17190 Salt, Spain

**Keywords:** antitumor agents 1, ribonucleases 2, pharmacokinetic 3, RNA-targeted drugs 4

## Abstract

Approaches to develop effective drugs to kill cancer cells are mainly focused either on the improvement of the currently used chemotherapeutics or on the development of targeted therapies aimed at the selective destruction of cancer cells by steering specific molecules and/or enhancing the immune response. The former strategy is limited by its genotoxicity and severe side effects, while the second one is not always effective due to tumor cell heterogeneity and variability of targets in cancer cells. Between these two strategies, several approaches target different types of RNA in tumor cells. RNA degradation alters gene expression at different levels inducing cell death. However, unlike DNA targeting, it is a pleotropic but a non-genotoxic process. Among the ways to destroy RNA, we find the use of ribonucleases with antitumor properties. In the last few years, there has been a significant progress in the understanding of the mechanism by which these enzymes kill cancer cells and in the development of more effective variants. All the approaches seek to maintain the requirements of the ribonucleases to be specifically cytotoxic for tumor cells. These requirements start with the competence of the enzymes to interact with the cell membrane, a process that is critical for their internalization and selectivity for tumor cells and continue with the downstream effects mainly relying on changes in the RNA molecular profile, which are not only due to the ribonucleolytic activity of these enzymes. Although the great improvements achieved in the antitumor activity by designing new ribonuclease variants, some drawbacks still need to be addressed. In the present review, we will focus on the known mechanisms used by ribonucleases to kill cancer cells and on recent strategies to solve the shortcomings that they show as antitumor agents, mainly their pharmacokinetics.

## 1. Introduction

The use of proteins as therapeutic agents has exhibited great potential to fight various diseases and particularly cancer. In comparison with genetic drugs, proteins are better targetable and thus regulate more specifically the biological processes, reducing side effects [1,2]. Moreover, the use of proteins as anticancer agents avoids permanent or random genetic alteration, off-target effects due to persistent gene expression, and the risk of carcinogenesis [2,3,4]. Secretory ribonucleases (RNases) are among these proteins with antitumor activity showing different mechanisms to destroy cancer cells. The last 20 years have witnessed a significant amount of work devoted to the design of novel RNases with improved antitumor properties, based on the knowledge gained from the mechanisms that the natural cytotoxic RNases present. Nevertheless, these non-genotoxic drugs still have some important drawbacks to overcome. In this review, we describe the known molecular bases of the cytotoxic action of these enzymes, which go further for their RNA degrading activity, and we discuss the strengths and challenges associated with their use as antitumor agents.

## 2. Secretory Ribonucleases Display a Vast Array of Functions

Secretory RNases are a vast group of enzymes that were initially considered as mere non-specific degradative proteins and that today are known to participate in a wide spectrum of biological activities (a very well-described review of these activities has been recently provided by Gotte and Menegazzi [5]). These RNases, found in eukaryotic and prokaryotic organisms, are secreted or accumulate inside cellular structures associated with secretory pathways, and therefore they can be found in spaces that are not normally associated with the presence of RNA. Secretory RNases belong to three different groups: pancreatic-type RNases, that are found in different vertebrate species, T1 RNases, that exist only in bacteria and fungus, and T2 RNases, that are present in organisms across kingdoms (for a review, see [6]).

In plants, characterized secretory RNases belong to the RNase T2 family and are implicated in mediating stress responses to different abiotic and biotic factors such as wounding, pathogen invasion, or P-starvation [7,8]. In addition, they are also implicated in housekeeping roles such as recycling rRNA [9], biosynthesis of tRNA-derived small-RNAs that participate in posttranscriptional regulation of gene expression [10], or maintaining self-incompatibility in pollination [11,12] (for a review, see MacIntosh and Castandet [13]). In microorganisms, T2 RNases are involved in different functions such as membrane permeability [14], scavenging of phosphate from RNA [15], perturbing the immune system [16], or controlling biofilm formation [17].

Finally, in vertebrates, apart from the digestion of rumen bacteria RNA, displayed by enzymes such as RNase A, pancreatic-type RNases display multiple activities. Some of them promote the suppression of certain cells, as is the case of the eosinophil-derived neurotoxin (EDN) that displays neurotoxic activity [18,19] or bovine-seminal RNase (BS-RNase) that has immunosuppressive, embryotoxic, and aspermatogenic activities [20,21]. Others promote cell survival activities such as the angiogenesis activity displayed by angiogenin [22,23] and RNase 4 [24] that further promotes neuronal survival under stress [25]. Among this group, several RNases are also implicated in the host defense against pathogens. For example, EDN displays chemoattractant effects that modulate the immune response [26] and human RNaseT2 has a role in regulating the innate immune response after bacterial challenge [27]. EDN also has antiviral activity against respiratory syncytial virus [28]. Eosinophil cationic protein (ECP) or RNase 3 presents antiviral [29,30] and bactericidal activity [31]. RNase 6 [32,33], RNase 7 [34], and RNase 8 [35] also display this latter activity. ECP exerts also cytotoxic activity against non-phagocytosable pathogens such as helminthic parasites [31]. Interestingly, different RNases have antitumor actions. Among them, we can mention bovine pancreatic RNase A [36], the oligomeric forms of BS-RNase [37,38], onconase (ONC) [39,40], and amphinases [41] from Rana pipiens, sialic acid-binding lectins (SBL) from *Rana catesbeiana* and *Rana japonica* [42,43], human RNASET2 [44], *Aspergillus niger* ACTIBIND T2 RNase [45], and different T1 RNases of microbial origin such as barnase and binase [46].

## 3. Mechanisms of Antitumor Action of Secretory RNases

The mechanisms by which these RNases induce the death of human tumor cells are diverse and, although not fully understood, for some of them, the RNase activity is not required. The most widely accepted mechanism of the cytotoxic action for most of these RNases, partially proved by many studies, consists of a series of steps (for a review, see Benito et al. [47]) that initiate when these RNases bind to tumor cell membranes and are internalized. Then, at some point, they translocate to cytosol where they evade mammalian protein RNase inhibitor (RI) and degrade RNA leading to apoptosis. The primary interaction of these RNases with the membrane cell is, for some of them, nonspecific, produced through electrostatic attraction between opposite charged groups. Since the membrane of tumor cells is more negatively charged than that of normal cells [48], the capacity of interaction with the former is higher for these basic proteins. This is a first mechanism that explains the selectivity for cancer cells. This mechanism is not, however, general and some of these RNases require the presence of a cell receptor. As examples, we can mention a still unidentified sialilated receptor specific of cancer cells, detected in different animal cancer cell lines and in the human gastric cancer MKN45 cell line, required for the SBLs’ entry into them [49] and surface actin present in cancer cells as the anchorage point of ACTIBIND T2 RNase [45]. The presence of a specific receptor for some of these RNases, such as ONC, is still controversial [50,51].

Finally, the interaction of BS-RNase to the cellular membrane seems to be facilitated by sulfhydryl–disulfide interchange between cell surface sulfhydryl’s and the inter-subunit disulfides that form the MxM dimer [52].

Not all the antitumor RNases need to internalize to exert their cytotoxic action. It has been described that *Bacillus pumilus* RNase (binase) exerts its cytotoxic effect on a SV40-transformed lung tumor cell line and that non-transformed cells are insensitive to this RNase [53]. It has been shown that binase does not penetrate SV40-transformed MLE-12 cells, but they exhibit high sensitivity to the RNase [53]. In addition, RNase A displays antitumor and antimetastatic activities that are associated with a decrease in serum miRNAs and an increase in tumor miRNAs [54] (see below).

Once cytotoxic RNases are internalized, their precise intracellular route to a compartment where the RNA is accessible may also explain the specificity of some of them for cancer cells. This is the case hypothesized for BS-RNase, which is localized in the trans-Golgi network of malignant but not normal cells [55]. After their membrane translocation, most of these RNases act on the cytoplasm cleaving the RNA although some of them also localize at least in part in the nucleus. These are the cases of BS-RNase, which has also been localized in the nucleolus [55], and binase [53]. Nuclear and more specifically nucleolus localization is very interesting since this sub-compartment is free of RI [56,57]. RI is a cytosolic protein of 50 kDa protein that tightly binds to some RNases inhibiting their activity [58] and therefore acting as a safeguard against extracellular RNases. Indeed, antitumor RNases by-pass the action of RI.

**Figure 1:**
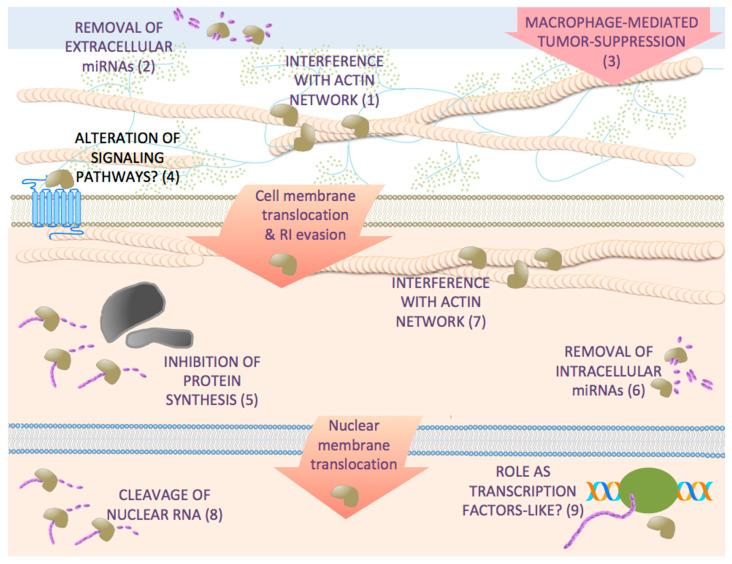
Mechanisms of the cytotoxic action of secretory RNases. Different RNases act in the extracellular space, in the cytosol and even in the nucleus. On the extracellular space, RNases like RNASET2 interfere with the organization of extracellular actin network, inhibiting cell motility and invasiveness (1). In other cases, they decrease the circulating miRNAs attenuating in part the tumor malignancy (2). It is also hypothesized that some of them, like RNASET2, could be a regulator of several functional features within the immune system inducing the macrophage mediated tumor suppression (3). They may also potentially function as ligands for different cell receptors that can or cannot drive their internalization or affect signal transduction pathways (4). Some of the RNases can translocate into the cytosol and evade the RI. There, some of them are able to inhibit protein synthesis by cleaving rRNA, tRNA, or mRNA (5). It is demonstrated that some RNases, like ONC, also can cleave miRNAs and precursors (6). RNases like ACTIBIND T2 disrupt intracellular actin filament and actin-rich extracellular extrusion organization inhibiting cell migration (7). Finally, some RNases reach the nucleus where they can cleave nuclear RNA (8) or potentially function as transcription factors-like (9). Because of all these activities, the RNases alter the pool of cytosolic and nuclear coding and non-coding RNAs inducing cell death.

As stated above, some RNases display antitumor activity independently of their enzymatic activity. It has been shown that ACTIBIND T2 RNase and its denatured form have an anticlonogenic activity on human cancer cell lines and inhibit xenograft tumor development in mice [45]. This protein acts by interfering with the organization of intracellular actin network, inhibiting cell motility and invasiveness [45]. This is also the case of its human homologue RNASET2. It is described that inactivation through mutation or by denaturation of human RNASET2 does not suppress its antitumor activity, indicating that this protein also has a catalytic-independent role in tumor suppression [44,59]. This protein disrupts intracellular actin filament and actin-rich extracellular extrusion organization in colon and melanoma cell lines inhibiting cell migration [60]. Furthermore, RNASET2 displays a non-cell autonomous oncosuppressive role altering the balance between the pro- and anti-tumor roles of the innate immune system through acting as an alarmin-like macrophage-mediated tumor suppressor gene [61]. Figure 1 shows a schematic representation of the different mechanisms of antitumor secretory ribonucleases.

## 4. Regulatory RNAs are Key Targets for Different Antitumor RNases

Once inside the cell, most of the cytotoxic RNases cleave and alter the balance of different types of RNA (Figure 1) even if these molecules interact with proteins forming ribonucleoprotein complexes that protect them. Initially, the antitumor properties of the RNases belonging to the vertebrate RNase A family were attributed to their ability to cleave RNA molecules directly involved in protein synthesis, precluding this process and inducing cell cycle arrest and apoptosis. BS-RNase and cSBL cleave rRNA [62,63] leading to inhibition of protein synthesis; however, in the last few years, it has become evident that the RNases-induced cell death shows features different from an indiscriminate translation inhibition.

The first evidence that the inhibition of protein synthesis through RNA degradation is not the only cytotoxic mechanism of vertebrate RNase A superfamily members was early described for ONC [64], with the report of an up- or downregulation of genes coding for proteins involved in cell cycle control and transcription factors [65,66]. It was described that ONC targets rRNAs [50] and tRNAs [67] but also mRNAs [65,68] and microRNAs as well as their precursors [69,70,71], which results in changes in the expression of multiple genes, either promoting their up- or downregulation. These changes can explain the different apoptotic [72,73,74,75] or autophagy [76] processes triggered by ONC although its efficiency and the effects produced in the cells were described as dependent on the molecular signature of each tumor cell [77] and thus cell-specific (see below). The global changes in the expression profile of miRNAs produced by ONC on treated cells have also been studied using microarray analysis. In three malignant mesothelioma (MM) cell lines, it has been observed that, from all the miRNAs analyzed, five showed increased expression while 15 showed decreased expression. However, this result was only corroborated by PCR analysis for miR-17* (3-fold upregulated) and miR-30c (0.4-fold downregulated) [78]. In a latter work, in MSTO-211H mesothelioma cells, ONC decreased the level of almost all studied miRNAs. Although again the results could not be corroborated by PCR [71], they allowed for proposing that miRNA precursors are an ONC substrate. Nevertheless, the use of a miR-17* mimic and a miR-30c inhibitor significantly decreased the ONC activity and the expression of NFKB1(p50) and its downstream targets [78]. This result is in agreement with other works that describe a decrease of NFKB1(p50) [69] and a downregulation in the steady state and subcellular distribution of NF-kappaB [66,79,80] as a result of ONC action on the treated cells.

Similar studies of genome-wide profiles of miRNAs in the tumor of mice bearing Lewis lung carcinoma (LLC) have been performed, after treatment with RNase A, by high-throughput Sequencing by Oligonucleotide Ligation and Detection (SOLiD^TM^) sequencing technology and RT-qPCR by Mironova et al. [54]. The results show an increase of upregulated miRNAs in the tumor tissue of treated animals compared to untreated control groups (116 were significantly upregulated and only seven downregulated of the 123 out of 615 tumor-derived miRNAs that showed level alteration). These results were verified by stem-loop qPCR. The results also show a significant decrease of miRNAs in the bloodstream. From these results, the authors hypothesized that RNase A would attenuate in part the tumor malignancy by altering the profile of blood oncogenic miRNAs. It is known that increased levels of bloodstream circulating RNAs and miRNAs that are originated in the tumors accompany tumor progression. Most of these tumor miRNAs circulate in the blood as stable complexes with Ago2 [44] protecting miRNAs from degradation. Since RNase A does not cleave the miRNA/Ago2 complex in vitro, authors hypothesize that RNase A could cleave different non-coding RNAs in the bloodstream generating a set of short fragments that would compete with miRNAs for binding with Ago2. This would lead to a displacement of miRNAs from miRNA/Ago2 complexes allowing the degradation of these miRNAs by RNase A. Among the tumor derived upregulated miRNAs, there were miRNAs with either tumor suppressor or oncogenic activity. Thus, it is difficult to unambiguously ascertain that the effects, described in previous works of the same group, on the reduction of tumor growth in mice bearing LLC after RNase A treatment [36], are due to the observed changes in the miRNA profile. Nevertheless, among the 100 top upregulated tumor-derived miRNAs, the authors found 11 of 12 miRNAs of the let-7 family, which are described to slowdown tumor progression and that are important for cancer patient survival [81,82]. The effects of RNase A treatment were attributed to its global action on the miRNA population. In addition, in the same work, the authors found, from the tumor-derived tissue samples, that the expression of some genes encoding key molecules known to be important for miRNA processing were upregulated in response to RNase A treatment. This led them to propose that RNase A mediates the enhancement of mature miRNAs expression being a beneficial event that confers to RNase A its tumor-suppressive function. In the same paper, they demonstrate with in vitro experiments (cell culture of primary LLC) that the RNase A activity is necessary for the boost of miRNAs in the tumor tissue, comparing the effect of the RNase A with that of DEPC-treated enzyme on the miRNA levels in LLC in culture, measured by stem-loop RT-qPCR. This result is surprising because, once inside the cell, wild type RNase A is inhibited by RI [47,83], and the amount of RNase A used in the experiment does not seem enough to saturate it, taking into account the endocytosis efficiency described for other cytotoxic members of the family [84]. As described for angiogenin (ANG) [85] (see below), the authors also proposed that RNase A could act as a transcription factor affecting the expression of miRNA processing genes and/or that of miRNAs.

## 5. Effects of Antitumor RNases on Target Cells beyond RNA Degradation

A very interesting aspect of RNases is their potential function as transcription factors-like and as ligands for different cell receptors that can or cannot drive their internalization (Figure 1). These roles may be important to understand how the members of the family that show a very reduced or null ribonucleolytic activity carry out their functions. The RNase A superfamily is classified in two subgroups, canonical (RNases 1-8) and non-canonical (RNases 9-13) [86]. The latter subgroup is deficient in RNase activity and, although to date little is known about their physiological functions, it is believed that they may play a role in host defense similar to other canonical members, such as EDN or ECP (for a review see [87,88]). Taking into account the omics results known to date on the cytotoxic action of RNases, it is likely that some members could behave as transcription factors-like or as cell-specific ligands that transduce signals to the target cells ultimately affecting the expression of different coding and/or non-coding RNAs (Figure 1). These roles are well defined for ANG, which presents a ribonucleolytic activity toward model substrates that is 10^−5^–10^−6^-fold lower than that of RNase A [89]. To date, different proteins have been suggested as receptors for ANG [90,91]. Among them, a putative 170 kDa cell surface protein was earlier proposed [92], which recently has been identified as Plexin-B2 in a variety of tumor and non-tumor cells as well as normal and malignant stem and progenitor cells [93] and, also, as epidermal growth factor receptor (EGFR) in pancreatic cancer cells [94]. In this latter work, analysis was performed by RNA-deep sequencing and demonstrated a global pattern of transcriptional changes induced by ANG treatment similar to that of EGF with a high percentage of overlap (∼80%) of affected genes. Gene Set Enrichment Analysis (GSEA) further showed that both stimuli, ANG and EGF, significantly enriched several gene signatures. Thus, ANG elicits signaling events that resemble EGF to control gene transcription in pancreatic cancer cells [94]. Moreover, previously, by ChIP-on-chip assay, it was described that a total of 699 genes, which are significantly associated with tumorigenesis, may be regulated by ANG [95].

This mechanism could be theoretically extended to other members of the RNase A family. Interestingly, it has been found recently that RNase A, but surprisingly not its homologous, human pancreatic RNase (HP-RNase), is a ligand of EGFR as ANG. It would be interesting to know whether other members of this family can act through cellular receptors because the identification of RNase receptors could provide novel molecular targets for intervention in cancer therapy.

## 6. Antitumor RNases Exert Pleiotropic Effects on Cancer Cells

A common observation for many of the results presented above is that many RNases affect multiple important pathways such as survival, proliferation, invasion, or migration. Microarray-derived transcription profiling experiments, corroborated by RT-qPCR, have demonstrated that the ONC upregulation of the transcription regulator, activating transcription factor 3 (ATF3), plays a central role in the key events elicited by this enzyme in ovarian treated cancer cells. ATF3 is networked to other transcription regulators that together can explain the effects of ONC on cell growth arrest [96]. In addition, in ONC- treated ovarian cancer cells, the MAPK and the JAK-STAT signaling pathways, both promoting tumorigenesis, were negatively affected [96]. Interestingly, the mechanism mediated by ATF3 is described as cell-type independent since the altered genes and the negative regulation of the JAK-STAT signaling pathway in ovarian cancer cell lines [96] are also found equally changed in different MM cell lines, derived from pleural tumors [68]. ONC also presents a very interesting antiviral activity [97,98,99,100], beyond RNA degradation, that is again mediated by ATF3 [96].

Mironova et al., using SOLiD^TM^ sequencing technology validated by RT-qPCR, have recently carried out a whole transcriptome analysis of the murine LLC after treatment of the tumor-bearing mice with RNase A [101] with a regime of i.m. enzyme injection as in a previous work on miRNA profiling [54]. The results show that many of the differentially expressed genes belong to metabolic pathways involved in carbohydrate, lipid, fatty acids, amino acids, and nucleotide metabolism with an increase in oxidative phosphorylation (OXPHOS) after the RNase A treatment, which is considered evidence of the reversal of the cancerous phenotype [102]. A similar result was previously published for an engineered HP-RNase, named PE5, that has acquired its cytotoxic properties because it is nuclear-directed, thus evading the action of RI [84,103]. The microarray-derived transcriptional profiling of PE5 regulated genes on the NCI/ADR-RES ovarian cancer cell line, corroborated by RT-qPCR analyses, shows that this nuclear-directed RNase (ND-RNase) causes pleiotropic effects. Among them, the downregulation of multiple genes that code for enzymes involved in deregulated metabolic pathways in cancer [104] such as those described as affected by RNase A in the in vivo RNAseq assay is remarkable. Moreover, RNase A negatively regulates multiple tumor-promoting pathways, including PI3K/AKT, TFG-β, JAK-STAT, and the canonical WNT signaling pathways [101]. Some of them are also affected by PE5 likely through the downregulation of oncogenes and upregulation of tumor suppressors, which certainly induce pleotropic effects that affect processes such as apoptosis and cell proliferation, angiogenesis, and invasiveness and metastasis, but that remarkably revert the metabolic deregulated pathways in cancer cells [104]. It is also worth mentioning that ONC also affects the MAPK and the JAK-STAT signaling pathways both in ovarian [96] and MM [68] treated tumor cell lines. All of the pathways mentioned control multiple cell processes, thus it is very difficult to find a general and straightforward relationship between the effects of these three RNases on the signaling pathways and their selective mechanism of tumor cell killing. Although it is interesting to realize that the studied RNases produce changes on the same signaling pathways, the final main affected cell processes may be similar or different depending on the RNase, at least from the present results. As described above, when comparing the effects of RNase A (in vivo) and those of PE5 (in vitro) on different type of tumors or tumor cells, a primary effect on reverting metabolic pathways deregulated in cancer is found. However, different effects are observed when comparing the results of whole transcriptome analysis by microarray technology on ovarian cancer cells treated with ONC, that acts in the cytosol, with those of PE5 that performs in the nucleus [96]. Very likely, depending on the RNase, where it acts in the cell and on the tumor signature, the final effects may be different although likely signaled by the same pathways. To blur further the scenario, the effects of the RNases on the non-coding RNAs and/or their precursors have to be taken into account. At present, as far as we know, the single results that show correlation are those related to the effects of RNase A on the genes involved in the biogenesis of miRNAs and the let-7 family of miRNAs. The whole transcriptome analysis of the LLC tumor-bearing mice after treatment with RNase A [101] shows a downregulation of genes encoding suppressors of miRNA biogenesis, some of them preventing the terminal processing of the let-7 family of miRNAs. This is in agreement with the studies of genome-wide profiles of miRNAs of mice bearing the same tumor, which show an upregulation of the let-7 family of miRNAs [54]. In addition, miRNA-494 binds to the 3′UTR of ATF3 and suppresses the transcription of this factor. In mice, its overexpression significantly attenuates the levels of ATF3 [105]. Although this miRNA is not described as directly downregulated by any of the studied RNases and in particular not by ONC, this could be an example of a link between the effects of RNases on miRNAs and the changes observed in the expression of different genes after treatment.

Although experimental data on the antitumor action of the RNases are accumulating, they are still insufficient for identifying a general way of cell response on the toxic and tumor-selective action of these drugs. The use of methodologies such us cDNA and miRNA microarrays, RNAseq and miRNAseq profiling, proteomics and phospho-proteomics, as well as the study of the RNases internalization pathways and the structures of the complexes between RNases and their receptors, surely will help to elucidate the complex mechanisms of RNases action and assist in their use as antitumor drugs, either alone or as coadjuvant in different regime therapies.

## 7. Natural and Modified RNases as Antitumor Drugs: Concerns and Opportunities

Many recombinant antitumor RNase variants have been generated over the last 20 years (for a review, see [83,106]). These variants either enhance the natural antitumor activity of their parental proteins or endow non-cytotoxic RNases with this activity. Among the protein antitumor drugs, RNases have opened a new way to tackle the cancer phenotype. Unlike classical antitumor therapies, they are not genotoxic drugs. Radiotherapy or chemotherapy inhibit tumor and normal cell proliferation by affecting DNA replication producing toxic effects and even inducing secondary tumors. On the other hand, RNases alter the gene expression and regulation either directly by RNA degradation or by acting as transcription factors-like or ligands of cell receptors resulting in multiple targeting. Other protein therapies directed to a single cell target are highly specific but sometimes cannot cope with the multifactorial nature of cancer. The multitarget effect of the RNases can hamper the appearance of drug resistance although at a risk of a certain loss of selectivity.

The efficacy of RNases as antitumor drugs is also limited by some characteristics intrinsic to their protein nature. As proteins, they present a remarkable difficulty to cross the biological barriers, such as the gastrointestinal mucosa or the cell membrane. On the other hand, they are small size proteins showing short half-lives in sera due to proteases digestion and rapid renal filtration, requiring multiple administrations and the use of high doses to reach the necessary concentration in tumors. This administration regime results in an accumulation of the protein in the kidneys leading to renal toxicity [107]. This is the case of ONC, which has reached phase III clinical trials as antitumor agent [83,108], that despite it being a poorly immunogenic RNase, causes damage of proximal kidney tubular cells due to a highly unspecific uptake in this tissue. This nonspecific uptake leads to a dose-limiting renal toxicity that limits its clinical application [109,110,111,112]. Moreover, the cellular uptake rate of ONC is only slightly faster than that of fluid-phase, thus the rate of internalization of ONC limits its effectiveness [113]. Other RNases such as EVade^TM^ RNase or OshadiR have reached clinical trials but have not gotten over phase II for the treatment of different cancers. In the following sections, we will describe the approaches recently developed to by-pass these concerns.

### 7.1. Nanocarriers and Nanostructures to Strengthen the Efficiency of Antitumor RNases

As stated above, RNases are small proteins and, therefore, they are easily cleared from the sera but do not easily by-pass the successive physiological barriers. Even more, they can be cleaved by proteases from the bloodstream, tumor microenvironment or endo-lysosomal compartments. A promising strategy to ameliorate the pharmacokinetics, efficiency, and selectivity of antitumor RNases is the use of nanocarriers that indeed reduces the need for multiple high dose administrations, resulting in lower adverse effects [114] (Table 1). Along this line, Xu et al. [115] have conjugated hyaluronic acid (HA) to RNase A (RNase A-HA) and have complexed it with synthetic lipid-based nanoparticles. This RNase A-HA inhibits cell proliferation of CD44-overexpressing A549 cells in a dose-dependent manner because HA increases the electrostatic complexation with cationic lipidoid carriers and facilitates tumor cell targeting via interaction with CD44 receptor, which is overexpressed on many solid tumor cell surfaces [115].

RNase A has also been loaded inside a self-assembled heparin-Pluronic (HP) nanogel (HPR nanogels) to protect the protein against protease degradation and enhance its intracellular delivery. HPR nanogels are efficiently internalized into HeLa cells inducing the cell death in an RNase dose-dependent manner. However, in this case, RNase A was undesirably dropped-off to the extracellular environment before reaching its intracellular target [116]. In order to prevent the unwanted release from the nanocarrier, Vermonden and collaborators [117] covalently immobilized RNase A into methacrylate-derivatized anionic dextran (dex-MA) nanogels through disulfide bonds to release the RNase A into the reductive cytosolic environment. Moreover, the particle surface charge was reversed by coating it with the cationic polymer polyethyleneimine (PEI) to trigger their cellular uptake. Coating of the nanogels with PEI showed high uptake by MDA-MB 231 breast cancer cells and dose-dependent induction of apoptosis [117].

More recently, Chen and coworkers [1] developed a hypoxia-sensitive nanogel formed through the self-assembly of azobenzene (Azo) and β-cyclodextrin (βCD) grafted onto poly (l-glutamic acid)-graft-poly (ethylene glycol) methyl ether (PLG-g-mPEG). RNase A could be efficiently loaded into the supramolecular nanogels (nano-RNase) in mild aqueous conditions. The nanogels enhanced the cellular uptake of the RNase and, under hypoxic conditions, promoted its intracellular release. In vivo studies showed that nano-RNase significantly prolonged the stability of the RNase in the circulation. The nano-RNase also exhibited enhanced antitumor activity compared to free RNase in murine triple-negative breast cancer models with minimal systemic toxicity. When the nano-RNase was combined with a nanoformulation of vascular disrupting agents PLG-g-mPEG/combretastatinA4 (nano-CA4), the hypoxic environment of the tumors accelerated the release of RNase and obtained a tumor suppression rate of 91.7% [1].

The binase antitumor activity has also been enhanced by complexation with natural halloysite nanotubes (HNTs). Cytotoxicity of the binase immobilized on HNTs against tumor colon cells increased twice compared to that of the binase alone due to its perfect absorption by cells and longer release [118].

Finally, in another delivery strategy recently reported by Ding and collaborators [119], RNase A was loaded into a DNA origami-based nanoplatform. Cancer cell-targeting DNA aptamers were also integrated into these origami nanosheets in order to enhance its uptake efficiency. This delivery platform based on DNA origami/RNase A complex showed an efficient cellular uptake inside MCF-7 cells and enhanced cancer cell killing mediated by intracellular RNA degradation compared to free RNase A [119].

### 7.2. Modification of RNases to Increase Their Pharmacokinetics and Antitumor Potency

In addition to the formulations that use nanocarriers or nanostructures to efficiently deliver RNases to the target cells, described in the precedent section, genetic or chemical modification of RNases has also resulted in new variants with a better pharmacokinetic behavior or effectivity. One of the first approaches used was the fusion or conjugation of RNases with different antibodies, antibody derivatives [120,121,122], or antibody mimic molecules [123]. This is a field of intense research and the reader is addressed to the recent review [124] for more information.

ONC has been fused to the N-terminal domain of transferrin (TF) (rONC-TFn) in order to increase its selectivity for tumor cells and reduce its toxic side effects. The expression level of TF receptor (TfR) in normal cells is low but in tumor cells can increase up to 100-fold due to the high demand for iron in rapidly growing tumor cells [125]. rONC-TFn can bind to TfR and increase the cytotoxicity to the tumor cells compared to ONC. Conversely, the entry of rONC-TFn into normal cells is lower, showing that the specificity of ONC is significantly enhanced by fusing with TFn [126].

On the other hand, Chlorotoxin (CTX) and ONC have been linked by a disulfide bond to prepare a CTX ONC conjugate to treat malignant gliomas, a type of tumor with few treatment options and poor prognosis [127]. CTX is a scorpion derived small peptide that can selectively bind malignant gliomas through the cell surface matrix metalloproteinase 2 (MMP 2) and annexin 2 overexpressed in these tumors [128,129]. Although TM 601 (I^131^ labeled CTX) has been used in clinical trials as a drug to treat gliomas [130,131], it is not strong enough to induce apoptosis in tumor cells. Nevertheless, the CTX-conjugated ONC showed an improved antitumor effect on both cultured glioma cells and in a mouse model than the mixture of CTX and ONC [132].

Other recent approaches in the same direction can be exemplified by the modification of RNase A with the pH-sensitive bifunctional AzMMMan linker and varying amounts of a histidine-rich cationic oligomer. These RNase A conjugates show an efficient intracellular delivery and controlled release without protein inactivation, which results in significant cytotoxicity for tumor cells [133].

Raines and collaborators [134] covalently conjugated a poly (ethylene glycol) (PEG) chain to RNase A to reduce its sensitivity to RI and increase its serum half-life. Although these conjugates showed a lower antiproliferative activity in vitro, PEGylation increased their serum half-life and nearly eliminated completely the tumor growth in a mouse xenograft model [134].

To increase the systemic circulation of the RNase A and hence its efficiency, Mo and colleagues [135] used a reduction-degradable polymeric crosslinked network around RNase A by means of a neutral monomer (acrylamide, AAm), a cationic monomer (*N*-(3-aminopropyl) methacrylamide, APMAAm), and a reduction-labile crosslinker (*N*,*N*’-bis(acryloyl) cystamine) (R-rNC) [135]. Treatment of the triple negative breast cancer cells MDA-MB-231 with R-rNC produces a rapid release RNase A within the tumor cells under the intracellular highly reductive conditions and cells are massively killed. In addition, they constructed a doxycycline (Doc) and R-rNC co-loaded nanocomposite (designated as Doc/R-rNC/aNG) to eliminate tumor cells and CSCs by combinational delivery. Doc/R-rNC/aNG has a polymeric nanogel core and a chemically conjugated HA shell. The polymeric core was formed by polymerization of three monomers, AAm, APMAAm and a synthetic azide-decorated monomer (AAm-N3), and an acid-cleavable crosslinker (glycerol dimethacrylate, GDA). The hierarchically-assembled nanocomposite showed superior cytotoxicity on MDA-MB-231 mammospheres and enhanced antitumor efficacy on xenograft tumor mouse model as a result of a prolonged systemic circulation, an increase of its tumor accumulation, a higher tumor perfusion, and an enhanced cellular uptake [135].

Crosslinking of the RNases to polymers has also been used to improve their delivery. RNase A has been linked through the genipin-mediated crosslinking of polyethylenimine (PEI) with an average molecular weight of 25 kDa (PEI25K), namely RGP [136]. The amino-rich structure of PEI provides a high positive charge density and promotes the cellular uptake through the electrostatic interaction with the negatively charged membrane surface of cancer cells. Moreover, it could also facilitate the endosomal escape through “proton sponge effect” [137,138]. The RGP nanoparticles were efficiently internalized in HeLa cells with a delivery efficiency of 97.9% and a 46.2% of cell apoptosis induction [136]. On the other hand, Ressler and coworkers [139] have reported that esterification of carboxyl groups of HP-RNase with a diazo compound allows its passage into the cytosol. After internalization, the nascent esters are hydrolyzed in situ by endogenous esterases. The process is traceless and allows for keeping the RNase activity and cytotoxicity of the enzyme, at least in the in vitro assays carried out [139].

Finally, the resistance of RNases to proteases degradation has been recently described for a cytotoxic variant of human pancreatic-RNase that has been modified through site-directed mutagenesis to increase its half-life [140]. Researchers have increased the thermal stability of this RNase around 17 °C by introducing additional disulfide bonds. This RNase had previously shown to be selectively cytotoxic for tumor cells by introducing an engineered nuclear localization signal into their sequence [141]. However, these modifications produced an important decrease in their stability compromising their behavior in vivo. When the stability of this variant was increased by engineering the new disulfide bond, it showed higher resistance to proteolysis when incubated with proteinase K or with human sera, while maintaining the cytotoxic activity on OVCAR-8 and NCI-H460 cells [140].

## 8. Conclusions

Although many of today’s anticancer drugs target the DNA or proteins in tumor cells, the last 20 years have witnessed an exciting increase of potential antitumor drugs that target different types of RNAs, from mRNA to the vast array of non-coding RNAs. The main advantage of these drugs is their lack of genotoxicity. Secretory ribonucleases are among these drugs due to their natural ability to cleave RNA. They show pleiotropic effects since they can act on multiple RNAs. This fact avoids the drawbacks presented by antitumor drugs directed to a single target, which are very specific, but unfortunately often not strong enough to cope with the heterogeneity of tumors and easily generate resistance. The mechanisms of antitumor action of secretory ribonucleases are progressively unveiled but as research goes on one gets aware that some of them remain elusive. Thus, we need more efforts to gain a deeper understanding of the action of these enzymes on tumor and non-tumor cells, mainly when their mechanisms are not directly linked to their enzymatic activity. Nevertheless, the knowledge gained on the action of natural antitumor RNases has inspired the engineering of secretory ribonucleases to get improved anticancer drugs, some of which have reached phases II/III of clinical trials. A notably drawback presented by RNases is their pharmacokinetics related to their protein nature and small size, which promotes an easy elimination of the body. To bypass this weakness, in the last few years, different approaches have been proposed, most of them directed at the use of these enzymes with different nanocarriers. We firmly believe that the advantages of these enzymes as antitumor drugs together with an effective protection that improves their pharmacokinetic will allow the production of selective, non-genotoxic antitumor drugs that will be able to handle with the multifactorial cancer phenotype.

## Figures and Tables

**Table 1:** Examples of nanocarrier systems used to improve the pharmacokinetic properties of antitumor RNases.

Nanocarrier System	Targeted Receptor	RNase	Action Mechanism	Ref
Hyaluronic acid lipid-based NP	CD-44	RNase A	Tumor cell targeting Decrease excretion	[115]
Heparin-Pluronic nanogel	NS ^1^	RNase A	Protect against protease degradation Decrease excretion	[116]
Methacrylate-derivatized anionic dextran reversed by coating it with polyethyleneimine	NS	RNase A	Enhance its intracellular delivery Decrease excretion	[117]
PLG-*g*-mPEG	NS	RNase A	Enhance cellular uptake under hypoxic conditions Decrease excretion	[1]
Halloysite nanotubes	NS	Binase	Improve cellular uptake and release Decrease excretion	[118]
DNA origami-based nanoplatform	NS	RNase A	Enhance uptake efficiency Decrease excretion	[119]

^1^ NS: Non-specific internalization.
